# Alkali Lithosilicates: Renaissance of a Reputable Substance Class with Surprising Luminescence Properties

**DOI:** 10.1002/anie.201808332

**Published:** 2018-09-21

**Authors:** Daniel Dutzler, Markus Seibald, Dominik Baumann, Hubert Huppertz

**Affiliations:** ^1^ Institut für Allgemeine Anorganische und Theoretische Chemie Universität Innsbruck Innrain 80–82 6020 Innsbruck Austria; ^2^ OSRAM Opto Semiconductors GmbH Mittelstetter Weg 2 86830 Schwabmünchen Germany

**Keywords:** crystal structure, high-temperature chemistry, lithosilicates, luminescence, phosphors

## Abstract

A hitherto unknown synthetic access to alkali lithosilicates, a substance class first described by Hoppe in the 1980s, is reported. With the synthesis and characterization of NaK_7_[Li_3_SiO_4_]_8_, a new representative has been discovered, expanding the family of known alkali lithosilicates. Astonishingly, NaK_7_[Li_3_SiO_4_]_8_ and the already established alkali lithosilicates Na[Li_3_SiO_4_] as well as K[Li_3_SiO_4_] display unforeseen luminescence properties, when doped with Eu^2+^. Na[Li_3_SiO_4_]:Eu^2+^ exhibits an ultra‐narrow blue, K[Li_3_SiO_4_]:Eu^2+^ a broadband, and NaK_7_[Li_3_SiO_4_]_8_:Eu^2+^ a yellow‐green double emission upon excitation with near‐UV to blue light. Consequently, all of the investigated substances of this class of compounds are highly interesting phosphors for application in phosphor converted LEDs.

Over the last couple of years, the hunt for new alternative light sources to replace incandescent light bulbs has resulted in the creation of countless phosphors for the use in phosphor converted light‐emitting diodes (pc‐LEDs).^1^, ^2^, ^3^, ^4^ In these pc‐LEDs, a short wavelength emitting light source (UV to blue LED) is combined with additional converting phosphors, which emit light of longer wavelengths, to compose the desired color.^5^, ^6^, ^7^, ^8^ While narrowband blue emitting phosphors like AELi_2_[Be_4_O_6_]:Eu^2+^ (AE=Sr, Ba) with a small Stokes shift are of essential importance for UV‐pumped RGB pc‐LEDs,^9^ narrowband red emitting phosphors are needed in In_1−*x*_Ga_*x*_N based blue pumping pc‐LEDs. During the search for adequate phosphors, many examples regarding red phosphors showed that especially ternary and multinary alkaline earth nitrides represent suitable host structures for doping with Eu^2+^.^10^, ^11^, ^12^, ^13^ Therefore, scientific research mainly focused on these systems. As a result, phosphors like Ca[LiAl_3_N_4_]:Eu^2+^,^14^ Sr[Mg_3_SiN_4_]:Eu^2+^,^15^ M[Mg_2_Al_2_N_4_]:Eu^2+^ (M=Ca, Sr, Ba, Eu),^16^ Sr[LiAl_3_N_4_]:Eu^2+^,^17^ and Sr_4_[LiAl_11_N_14_]:Eu^2+^ 18 were found, which have been proven to exhibit surprising narrowband red emissions.

Interestingly, Ca[LiAl_3_N_4_] and Sr[Mg_3_SiN_4_] both crystallize in the Na[Li_3_SiO_4_] structure type,^14^, ^15^ and Sr[LiAl_3_N_4_] in the Cs[Na_3_PbO_4_] structure type.^17^, ^19^ The latter structure type is also shared by K[Li_3_GeO_4_] and K[Li_3_SiO_4_].^20^ This finding is highly uncommon owing to the fact that the above mentioned pure nitrogen compounds, represented by three‐dimensional tetrahedra networks, only rarely possess pure oxide species as isotypic counterparts. Ca[LiAl_3_N_4_]–Na[Li_3_SiO_4_] and Sr[LiAl_3_N_4_]–K[Li_3_SiO_4_] are two examples of these rare cases. Having the major structural similarities between these compounds in mind, it is more than astonishing that only little is known about mixed phases in this field.^4^

Based on these considerations, the question arose if there are alkali metal silicates or lithosilicates, which could be suitable for modern phosphor materials. To answer this question, several alkali metal silicates such as Na[Li_3_SiO_4_] and K[Li_3_SiO_4_], originally synthesized by Hoppe in the 80s and 90s, have been revisited.^20^, ^21^ Owing to the fact that this substance‐class only hosts monovalent cations, it was widely believed that their host structures would be unsuitable for doping with Eu^2+^.

In the following, we report on the three lithosilicate phosphors Na[Li_3_SiO_4_]:Eu^2+^, K[Li_3_SiO_4_]:Eu^2+^, and NaK_7_[Li_3_SiO_4_]_8_:Eu^2+^ (new structure type), which are the first examples of this substance class. Surprisingly, these novel phosphors exhibit extremely diverse luminescence properties, despite their structural similarities, while maintaining a relatively high quantum efficiency (QE) and low thermal quenching (TQ). Their emissions range from narrow blue to a broadband warm white, which we explain in terms of structure‐property relationships. In particular, NaK_7_[Li_3_SiO_4_]_8_ doped with Eu^2+^ shows a combination of narrowband green and broadband red emission upon excitation with blue light, making it a promising compound for application in pc‐LEDs.^1^

The original syntheses of Hoppe et al. started from the pure alkali oxides in closed Ni‐ampules. Herein, we report the syntheses of Na[Li_3_SiO_4_], K[Li_3_SiO_4_], and the novel compound NaK_7_[Li_3_SiO_4_]_8_ by solid‐state synthesis from the easy accessible alkali carbonates and SiO_2_ in an open system (see the Experimental Section). The crystal structures of Na[Li_3_SiO_4_] and K[Li_3_SiO_4_] were confirmed by powder diffraction. The structure of NaK_7_[Li_3_SiO_4_]_8_ was elucidated by single‐crystal X‐ray diffraction analysis (see below).

Na[Li_3_SiO_4_] crystallizes in the tetragonal space group *I*4_1_/*a* (no. 88) with *a*=1078.4(1) and *c*=1263.3(1) pm. The structure consists of endless chains of Na‐polyhedra (coordination number (CN)=7+1) along [001], interconnected via LiO_4_ and SiO_4_ tetrahedra, which for their part form the three‐dimensional anionic framework. K[Li_3_SiO_4_] on the other hand crystallizes in the triclinic space group *P* $\bar{1}$ (no. 2) with *a*=574.4, *b*=734.8, *c*=971.02 pm, *α*=83.5, *β*=76.6, and *γ*=79.9°. Two crystallographically distinguishable potassium sites form endless chains along [111] and, like in Na[Li_3_SiO_4_], LiO_4_ and SiO_4_ tetrahedra form the anionic framework. Although Na[Li_3_SiO_4_] and K[Li_3_SiO_4_] do not share the same crystal structure, they show significant structural similarities.^20^, ^21^ The new compound NaK_7_[Li_3_SiO_4_]_8_ crystallizes in a novel structure type in the tetragonal space group *I*4_1_/*a* (no. 88) with *a*=1555.57(8) and *c*=1274.71(7) pm.^22^ The compound shows a close structural relationship to the aforementioned compounds Na[Li_3_SiO_4_] and Ca[LiAl_3_N_4_]. In analogy, it can be described as an ordered variant of the UCr_4_C_4_‐structure type.^23^

The structure of NaK_7_[Li_3_SiO_4_]_8_ consists of a highly condensed network built up from SiO_4_ tetrahedra and distorted LiO_4_ tetrahedra. These tetrahedra form *vierer* ring^24^ channels alongside [001] by corner‐sharing. Each oxygen atom centers four different tetrahedra (3 LiO_4_ and 1 SiO_4_) leading to a degree of condensation of *κ*=1 (atomic ratio (Li,Si)/O). The mean Li−O (Ø=201.7(4) pm) and Si−O (Ø=163.9(2) pm) bond lengths are in good agreement with the sum of the ionic radii.^25^ The unit cell consists of two types of channels designated as CH1 and CH2 (Figure 1 a,b), which are distinguishable by their central cations (see below). Within the unit cell, every CH1 is surrounded by four CH2 and vice versa. All channels are interconnected by joint edges and vertices. The channels CH1 and CH2 assemble the anionic framework building up a third, empty channel (center of Figure 1 a). The first type of channel CH1 has a 4_1_ screw axis in its center. This screw axis is not aligned with the K1 position, leading to a slight offset of the central atom. Furthermore, each *vierer* ring in this channel consists of one SiO_4_ and three LiO_4_ tetrahedra. Owing to the 4_1_ screw axis, the SiO_4_ tetrahedra form a helix‐like structure along [001] (Figure 1 b/top). Therefore, six LiO_4_ and two SiO_4_ tetrahedra surround the K1 position. This asymmetric surrounding results in a variance of the K−O bond lengths from 262.5(2) to 287.6(2) pm.

**Figure 1:**
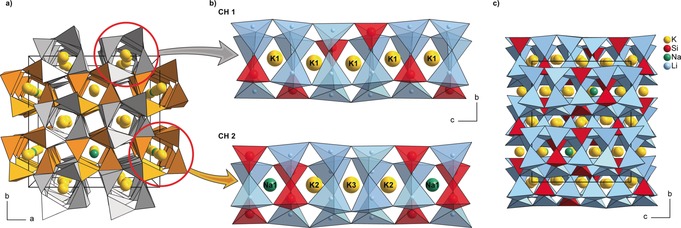
Crystal structure of NaK_7_[Li_3_SiO_4_]_8_. Figure 1 a shows the arrangement of the *vierer* ring channels along [00$\bar{1}$ ], whereby the K1‐strands (CH 1/Figure 1 b, top) are represented by the gray *vierer* ring channels and the Na‐K‐strands (CH 2/Figure 1 b, bottom) by the orange ones. Both strands are viewed in the [$\bar{1}$ 00] direction. Figure 1 c shows the complete structure alongside [$\bar{1}$ 00] with the LiO_4_ tetrahedra (blue), SiO_4_ tetrahedra (red), K atoms (yellow), and Na atoms (green).

The second type of channel CH2 contains three crystallographically distinguishable sites: K2, K3, and the Na1 site. In comparison to CH1, the channel symmetry is different. All of the cation sites are aligned along a $\bar{4}$ inversion axis with the cation K3 on the inversion center. There is an obvious preference from the SiO_4_ tetrahedra towards the sodium site, which is surrounded by four LiO_4_ and four SiO_4_ tetrahedra. This can be attributed to the smaller ionic radius of Na^+^. To compensate the size difference between a potassium and a sodium cation, the smaller SiO_4_ tetrahedra are located near the sodium site. For the same reason, the K2 site shows an asymmetry in its surrounding. The K^+^ ion is surrounded by two SiO_4_ and six LiO_4_ tetrahedra, dislocated from its central position away from the sodium cation and the two SiO_4_ tetrahedra. The K3 site is the only position solemnly surrounded by LiO_4_ tetrahedra (Figure 1 b, bottom).

In summary, the structure contains four sites (Na1, K1, K2, and K3) suitable for hosting an Eu^2+^ cation, which could serve as an activator ion for the luminescence. On all possible sites, Eu^2+^ would substitute a monovalent cation. The hollow space in the empty channels is too small to house Eu^2+^. Further details on the crystallographic data are given in the Supporting Information.

The investigations of the luminescence properties were carried out on powder samples for Na[Li_3_SiO_4_]:Eu^2+^ and K[Li_3_SiO_4_]:Eu^2+^, while for the novel compound NaK_7_[Li_3_SiO_4_]_8_:Eu^2+^ single‐crystals, with the cell parameters received from the structural refinement, were measured, to ensure that the measured properties originate exclusively from the determined structure. As excitation sources, near‐UV light (400 nm) for powders or blue light (460 nm) for single‐crystals were used. Na[Li_3_SiO_4_]:Eu^2+^ exhibits a narrow blue luminescence (*λ*_max_=469 nm/FWHM=32 nm). Compared to the known narrowband blue emitters SrSi_6_N_8_:Eu^2+^ (*λ*_max_=450 nm/FWHM=44 nm)^26^ and Sr_0.25_Ba_0.75_Si_2_O_2_N_2_:Eu^2+^ (*λ*_max_=472 nm/FWHM=37 nm),^27^, ^28^ the novel phosphor shows a narrower emission profile. The recently discovered material AELi_2_[Be_4_O_6_]:Eu^2+^ (AE=Sr, Ba) with *λ*_max_=454–456 nm exhibits an even smaller FWHM value of only about 25 nm.^9^ K[Li_3_SiO_4_]:Eu^2+^ shows a broadband, near‐warm‐white emission (Figure 2). Further information on the execution of the measurements, and the luminescence properties (thermal quenching, quantum efficiency and excitation spectra) can be found in the Supporting Information.

**Figure 2:**
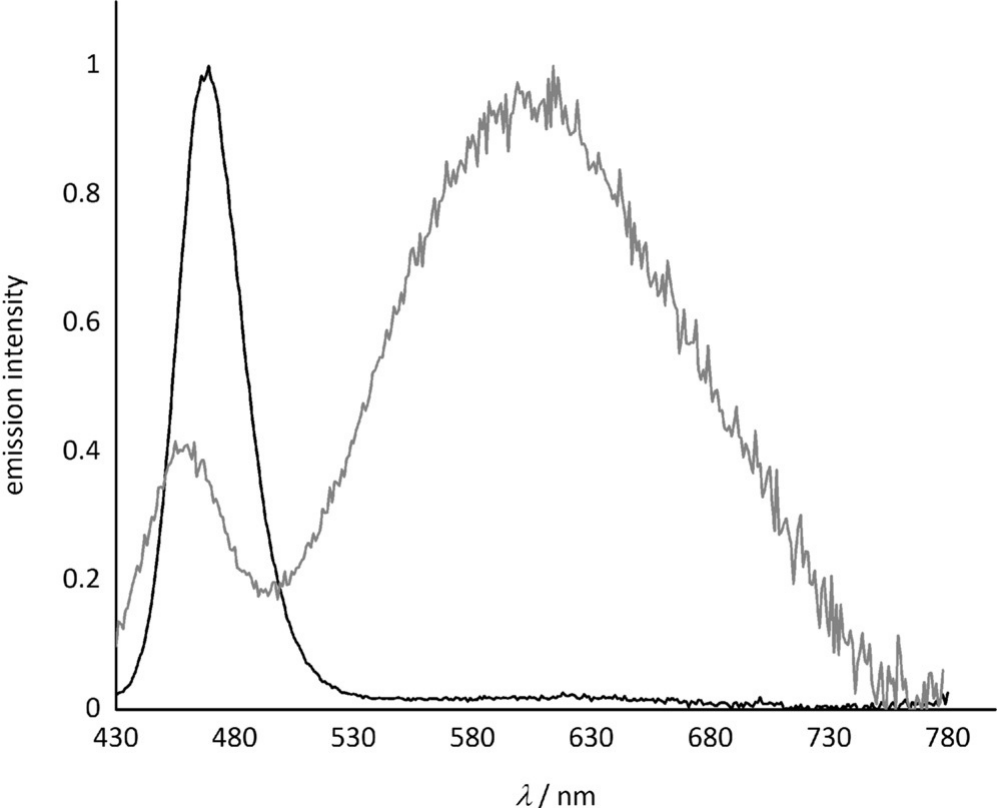
Powder emission spectra of Na[Li_3_SiO_4_]:Eu^2+^ (black) and K[Li_3_SiO_4_]:Eu^2+^ (gray) by excitation with near‐UV light (400 nm).

The substance NaK_7_[Li_3_SiO_4_]_8_:Eu^2+^ shows a bright‐green emission with a unique spectrum upon excitation at 460 nm (Figure 3). This spectrum consists of two different emission bands, the first peaking at 515 nm with an exceptionally small full‐width at half‐maximum (FWHM) of 49 nm, whereas the second exhibits a broad peak with a maximum at 598 nm and a FWHM of about 138 nm. Even though these are the first unoptimized samples of the new phosphors, they already show a QE of about 60 % and a favorable thermal quenching (TQ) behavior (Supporting Information, Figure S3).

**Figure 3:**
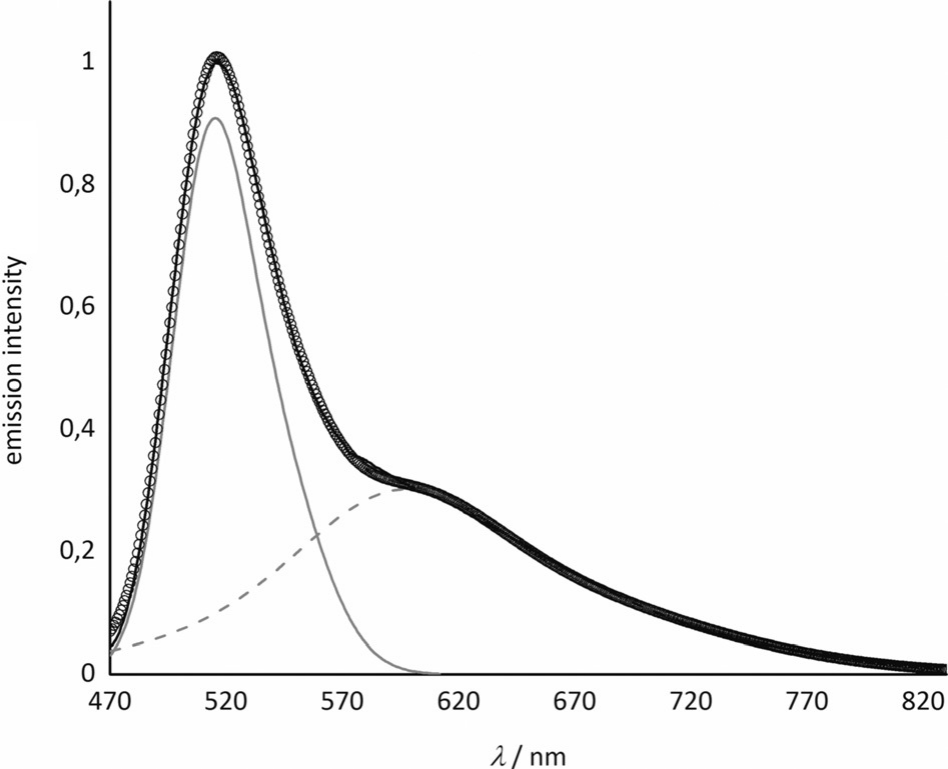
Single‐crystal emission spectrum of NaK_7_[Li_3_SiO_4_]_8_:Eu^2+^ (solid line, *λ*_exc._=460 nm) in combination with two Gaussian curves (gray) to describe the total emission (calculation, black circles) as a two peak band.

To understand the various luminescence properties, a closer look at the coordination of the possible Eu^2+^ sites is needed. Compared to the nitrogen‐coordinated Eu^2+^ sites in the nitrides Ca[LiAl_3_N_4_]:Eu^2+^ and Sr[LiAl_3_N_4_]:Eu^2+^, the surroundings of the possible doping sites in the three structures presented here show great similarities. In the first coordination sphere of the lithosilicates, these sites exhibit an eight‐fold coordination regarding O, with a varying degree of local symmetry. In the case of Na[Li_3_SiO_4_], there is only one Na position (coordination number (CN): 7+1) suitable for doping, whereas in K[Li_3_SiO_4_] there are two highly asymmetric coordinated K sites (both with a CN of 8), which could host Eu^2+^. An eight‐fold coordination can also be found for the four possible doping sites (Na1, K1, K2, and K3) in the compound NaK_7_[Li_3_SiO_4_]_8_, where the atoms Na1 and K3 have a symmetric cuboid coordination, while the atoms K1 and K2 exhibit a slightly more asymmetric coordination sphere. In total, we assume that the first coordination sphere of all potential doping sites is too similar to explain the highly diverse luminescence properties observed experimentally. Obviously, the influence of the extended coordination sphere on the doping sites has a significant effect on the luminescence properties. The extended coordination sphere of the alkali metal cations is quite complex, including silicon and lithium cations (Supporting Information, Figures S1 and S2). In a first approach, the coordination polyhedra towards the silicon cations are considered. In comparison to lithium, the Si^4+^ cations have a higher partial charge exhibiting a substantially better and more rigid positioning in the anionic framework. This presumably leads to a higher interaction with the central cation position. As can be seen from the Supporting Information, Figures S1 and S2, the coordination towards the lithium cations is much more complex and because of their higher mobility they are left out of consideration. The Na‐site in Na[Li_3_SiO_4_]:Eu^2+^ shows a tetrahedral coordination by Si^4+^, leading to the before mentioned ultra‐narrow blue emission band. In contrast, the second coordination sphere in K[Li_3_SiO_4_]:Eu^2+^, where both K‐sites show a five‐fold coordination by Si^4+^, yields a double banded spectrum with its main emission being an orange broadband one. In comparison with these two given phosphors, the luminescence properties of NaK_7_[Li_3_SiO_4_]_8_:Eu^2+^ lie somewhere in between those two phases. In the following, we give an explanation on how these properties are linked to the new structure type, and we propose that in this particular case the Eu^2+^‐luminescence is mainly influenced by the extended coordination sphere.

When compared to Na[Li_3_SiO_4_]:Eu^2+^, it can be assumed that the Na‐site in NaK_7_[Li_3_SiO_4_]_8_:Eu^2+^ is unsuitable for an occupation with Eu^2+^. A narrowband blue emission would be expected due to the tetrahedral coordination by Si^4+^ in the extended coordination sphere. The differences in the first and extended coordination sphere are not sufficiently significant to explain an emission shift from narrow blue to narrow green. Eu^2+^‐substitution on the K1‐site seems to be the most likely cause of the broadband orange emission. By comparison of the coordination of this site with the two K‐sites in K[Li_3_SiO_4_]:Eu^2+^, it is clear that they are quite similar. In NaK_7_[Li_3_SiO_4_]_8_:Eu^2+^, K1 shows an asymmetric cuboid coordination towards O, as well as a 4+1 extended coordination sphere (Si‐cations) resulting in an asymmetric trigonal bipyramid (Figure 4). Therefore, it is reasonable to assign this site to be responsible for the broadband emission of NaK_7_[Li_3_SiO_4_]_8_:Eu^2+^.

**Figure 4:**
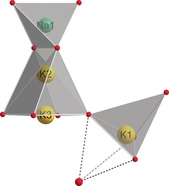
View of the extended coordination sphere of the monovalent cations regarding the silicon atoms (red) in NaK_7_[Li_3_SiO_4_]_8_:Eu^2+^ with a view along [$\bar{1}$ 00].

Consequently, the narrowband green emission of NaK_7_[Li_3_SiO_4_]_8_:Eu^2+^ should derive from one of the two remaining sites, the K2 or the K3 position. Both sites exhibit a cuboid (K3) or a slightly deformed cuboid coordination (K2) towards oxygen. The bond lengths lie between 272.0(2) and 289.8(2) pm, which is an adequate space for an Eu^2+^ cation. In both cases, the extended coordination sphere (Si cations) is not comparable to the coordination of the cation sites in Na[Li_3_SiO_4_] or K[Li_3_SiO_4_]. K2 shows a trigonal prismatic and K3 a square planar surrounding by Si^4+^ (Figure 4). Therefore, both sites could act as the doping site, but it is reasonable to assume that only one of those is favored, otherwise a broader or even split emission profile should be observed. Unfortunately, an exact localization of the luminescence center is not possible.

In all of the here presented novel phosphors, Eu^2+^ replaces monovalent cations. To ensure charge neutrality, vacancies on the highly mobile Li sites, resulting in a local variation of the Li/Si distribution in the network, or vacancies on the monovalent cation sites are needed. Because of the low degree of substitution, these effects withdraw themselves from detection via single‐crystal X‐ray diffraction and most other analytical methods.

In conclusion, a new, previously overlooked member of the family of alkali lithosilicates, namely NaK_7_[Li_3_SiO_4_]_8_, is introduced. The compound shows a novel structure type with a highly condensed network of LiO_4_ and SiO_4_ tetrahedra containing monovalent cations positioned in *vierer* ring channels along [001]. Na[Li_3_SiO_4_]:Eu^2+^, K[Li_3_SiO_4_]:Eu^2+^, and the new compound NaK_7_[Li_3_SiO_4_]_8_:Eu^2+^ represent the first three members of a novel phosphor‐substance‐class, where purely monovalent cations could be partially substituted with Eu^2+^, resulting in unexpected luminescence properties not foreseen for this substance class. Na[Li_3_SiO_4_]:Eu^2+^ shows a ultra‐narrow blue emission *λ*_max_ at 469 nm with a FWHM of 32 nm. According to LED simulations, K[Li_3_SiO_4_]:Eu^2+^ and NaK_7_[Li_3_SiO_4_]_8_:Eu^2+^ both could be used in single phosphor white pc‐LEDs. K[Li_3_SiO_4_]:Eu^2+^ in combination with a near‐UV LED yields a warm white emission with a color point close to the Planckian locus at 2700 K and high color rendering index (CRI>80, R9>0). NaK_7_[Li_3_SiO_4_]_8_:Eu^2+^ on the other hand could result in a cold‐white single phosphor pc‐LED with a CCT higher than 8000 K and high color rendering index (CRI>80, R9>0).

Furthermore, by comparing the properties of the novel phosphor NaK_7_[Li_3_SiO_4_]_8_:Eu^2+^ to the luminescence of simpler structures like Na[Li_3_SiO_4_]:Eu^2+^ or K[Li_3_SiO_4_]:Eu^2+^, structure property relations regarding the luminescence could be given. Consequently, by revisiting an old substance class with new or improved methods, it could be shown that alkali lithosilicates hold the potential for application in pc‐LEDs. Owing to the straightforward and comparably inexpensive synthesis method, these new materials could even break the supremacy of alkaline‐earth based pure nitrides and oxo‐nitrides as the go‐to materials for phosphors.

## Experimental Section

The different compounds were synthesized by means of a conventional solid‐state reaction in a tube‐furnace at 1000 °C. As starting materials various mixtures of Na_2_CO_3_, K_2_CO_3_, Li_2_CO_3_, SiO_2_, and 2 mol % Eu_2_O_3_ as a doping agent were used. The reduction from Eu^3+^ to Eu^2+^ was facilitated by a constant flow of forming‐gas (92.5/7.5) during the experiment. The obtained lithosilicate samples can be handled at ambient air. They are stable over weeks in the laboratory and can be heated to >200 °C without decomposition (Supporting Information, Figure S6). Furthermore, they are suitable for mixtures with silicone as a matrix material.

A more detailed description of the synthesis and further experimental details are provided in the Supporting Information.

## Conflict of interest

The authors declare no conflict of interest.

## Supporting information

As a service to our authors and readers, this journal provides supporting information supplied by the authors. Such materials are peer reviewed and may be re‐organized for online delivery, but are not copy‐edited or typeset. Technical support issues arising from supporting information (other than missing files) should be addressed to the authors.

Supplementary
