# Supplementary material for: Alkali Lithosilicates: Renaissance of a Reputable Substance Class with Surprising Luminescence Properties
Source: Angew Chem Int Ed Engl. 2018 Sep 21;57(41):13676–80. doi: 10.1002/anie.201808332 (PMC6197047; doi:10.1002/anie.201808332)
Supplement: Supplementary file 1 — Supplementary [file ANIE-57-13676-s001.pdf]

Angewandte Chemie
Eine Zeitschrift der Gesellschaft Deutscher Chemiker

Supporting Information

Alkali Lithosilicates: Renaissance of a Reputable Substance Class with Surprising Luminescence Properties

*Daniel Dutzler, Markus Seibald, Dominik Baumann, and Hubert Huppertz**

anie_201808332_sm_miscellaneous_information.pdf

Table of Contents

Experimental Procedures.. 2
Results and Discussion.. 3
References.. 8
Author Contributions.. 9

Experimental Procedures

Synthesis

According to Hoppe[1], powders and single-crystals of $NaLi_3SiO_4$ and KLi_3SiO_4 can only be obtained via a stoichiometric high-temperature reaction of the respective oxides in well-shut Ni-ampoules. As our experiments showed, the same results can be obtained via conventional solid-state reactions.

$Na[Li_3SiO_4]:Eu^{2+}$ was synthesized starting from a stoichiometric mixture of Na_2CO_3, (Merck, > 99.9 %), Li_2CO_3 (Merck, > 99.9 %), SiO_2 (Alfa Aesar, > 99.5 %), and for doping purposes 2 mol% Eu_2O_3 (OSRAM company). The starting mixture was thoroughly grounded in an agate mortar at ambient conditions and filled into a Ni-crucible. The open crucible was placed in a tube-furnace and fired at 1000 °C for 10 hours under a constant flow of forming gas (7.5 % H_2, and 92.5 % N_2).

$K[Li_3SiO_4]:Eu^{2+}$ was synthesized starting from a stoichiometric mixture of K_2CO_3, (Carl Roth, > 99 %), Li_2CO_3 (Merck, > 99.9 %), SiO_2 (Alfa Aesar, > 99.5 %), and for doping purposes 2 mol% Eu_2O_3 (OSRAM company). The starting mixture was thoroughly ground in an agate mortar at ambient conditions and filled into a Ni-crucible. The open crucible was placed in a tube-furnace and fired at 1000 °C for 4 hours under a constant flow of forming gas (7.5 % H_2, and 92.5 % N_2).

$NaK_7[Li_3SiO_4]_8:Eu^{2+}$ was synthesized starting from a mixture of Na_2CO_3, (Merck, > 99.9 %), K_2CO_3, (ChemPur, > 99.9 %), Li_2CO_3 (Fluka, > 99 %), SiO_2 (Alfa Aesar, > 99.5 %), and for doping purposes 2 mol% Eu_2O_3 (Treibacher, > 99.9 %). The starting materials were thoroughly ground in an agate mortar at ambient conditions and filled into a Ni-crucible. The open crucible was placed in a tube-furnace and fired at 1000 °C for 4 hours under a constant flow of forming gas (10 % H_2, and 90 % N_2). The novel compound first occurred in a non-stoichiometric reaction of Na_2CO_3, K_2CO_3, Li_2CO_3, and SiO_2 in a ratio of 1:1:6:4, which should have resulted in a compound with the stoichiometry of $NaK(Li_3SiO_4)_4$, however this composition was not realized. After identifying the unknown material via single-crystal diffraction, we were able to synthesize it in a stoichiometric reaction.

Undoped samples of all compounds could also be obtained by repeating the same synthesis in Pt-crucibles in a muffle furnace.

Single-crystal structural analysis

Small single-crystals of the phase $NaK_7(Li_3SiO_4)_8$ were selected from the sample using a polarization microscope. A D8 Quest Kappa diffractometer (Bruker, USA) equipped with a Photon 100 detector using monochromatic Mo$K_{\alpha 1}$ radiation (λ = 71.07 pm) generated by a microfocus X-ray tube (Incoatec, Germany) was used to collect the single-crystal intensity data at lower temperatures (-80 °C). SAINT[2] and SADABS[3] were applied for data processing and multiscan absorption correction. The structure was solved with SHELXS[4] (version 2014/1) and refined via SHELXL[5] (version 2013/4) using WINGX[6] (version 2014/1). The full-matrix least squares refinement against F^2 yielded R_1 = 0.0320, wR_2 = 0.0850, and GOF = 1.187. For detailed information on the refinement parameters and crystallographic data, such as positional parameters, anisotropic displacement parameters, and interatomic distances see Tables 1–4. Further information on the crystal structure investigation can be obtained from the joint CCDC/FIZ Karlsruhe deposition service on quoting the deposition number CCDC-1861384.

Luminescence

Luminescence Spectroscopy: The emission signal of $NaK_7(Li_3SiO_4)_8:Eu^{2+}$ was measured by exciting crystals showing the cell of $NaK_7(Li_3SiO_4)_8$ with a 460 nm laser (model Sapphire 460/10, 10 mW; COHERENT). The converted light was collected using a multi-mode optical fiber (QP 600-2-VIS/BX; Ocean Optics) and finally detected in a spectrometer (QE 65000; Ocean Optics). In order to determine the luminescence properties of $Na[Li_3SiO_4]:Eu^{2+}$ and $K[Li_3SiO_4]:Eu^{2+}$ powder samples, a HORIBA Fluoromax 4 spectrophotometer was used. The emission spectrum was measured in the wavelength range between 430 and 780 nm (step size 1 nm) using an excitation wavelength of 400 nm and an integration time of 0.2 seconds per step.

Results and Discussion

Crystal structure details

Table S1. Crystallographic and refinement data of $NaK_7[Li_3SiO_4]_8$

empirical formula	$NaK_7[Li_3SiO_4]_8$
molar mass, g mol^{-1}	1199.97
crystal system	tetragonal
space group	$I4_1/a$ (origin choice 2)
**Single-crystal data**	
radiation	MoK_α (λ = 71.07 pm)
a, pm	1555.57(8)
c, pm	1274.71(7)
V, nm^3	3.0845(4)
Z	4
T, K	193(2)
calculated density, g cm^{-3}	2.58
F(000)	2336
absorption coeff., mm^{-1}	1.432
absorption correction	multi-scan
R_σ, %	1.35
R_{int}, %	4.20
index range, *hkl*	±23; ±23; ±19
θ range, °	3.3–32.5
reflections total/unique	57717/2768
data/parameters	2768/163
R_1/wR_2 [$I \geq 2\sigma(I)$], %	3.20/8.50
R_1/wR_2 [all data], %	3.66/8.72
GOF on F^2	1.187
residual density, e Å^{-3}	+0.70/-1.47

Table S2. Wyckoff positions, atomic coordinates, and equivalent isotropic displacement parameters U_{eq} (Å^2 × 10^{-3}). U_{eq} is defined as one third of the trace of the orthogonalized U_{ij} tensor.

atom	Wyckoff position	*x*	*y*	*z*	U_{eq}
K1	16*f*	0.23953(3)	0.98699(3)	0.26441(3)	0.0125(1)
K2	8*e*	½	¼	0.60371(5)	0.0114(2)
K3	4*a*	½	¼	⅜	0.0160(2)
Na1	4*b*	½	¼	⅞	0.0141(3)
Si1	16*f*	0.35450(3)	0.32081(3)	0.74883(3)	0.0027(1)
Si2	16*f*	0.32027(3)	0.15076(3)	0.37804(3)	0.0041(1)
O1	16*f*	0.44450(8)	0.37555(8)	0.74454(9)	0.0052(2)
O2	16*f*	0.35279(9)	0.19704(8)	0.2697(1)	0.0076(2)
O3	16*f*	0.37603(8)	0.21769(8)	0.74898(9)	0.0057(2)
O4	16*f*	0.30553(8)	0.34900(9)	0.8570(1)	0.0068(2)
O5	16f	0.30167(8)	0.34542(9)	0.6420(1)	0.0071(2)
O6	16f	0.36469(8)	0.05416(8)	0.37574(9)	0.0055(2)
O7	16f	0.35848(9)	0.19738(8)	0.4835(1)	0.0071(2)
O8	16f	0.21592(9)	0.14266(10)	0.3823(1)	0.0100(2)
Li1	16f	0.3353(2)	0.3188(2)	0.5002(2)	0.0108(6)
Li2	16f	0.5645(2)	0.3991(2)	0.7506(2)	0.0082(5)
Li3	16f	0.1682(2)	0.1825(2)	0.2524(3)	0.0101(6)
Li4	16f	0.4287(3)	0.4330(2)	0.8779(3)	0.0129(6)
Li5	16f	0.4099(2)	0.9390(2)	0.3839(3)	0.0123(6)
Li6	16f	0.3128(3)	0.1785(3)	0.6233(3)	0.0236(9)

Table S3. Anisotropic displacement parameters U_{ij} (Å^2 × 10^{-3}) of $NaK_7[Li_3SiO_4]_8$ (standard deviations in parentheses).

atom	U_{11}	U_{22}	U_{33}	U_{23}	U_{13}	U_{12}
K1	0.0136(2)	0.0106(2)	0.0134(2)	-0.0005(2)	-0.0048(2)	0.0017(2)
K2	0.0101(2)	0.0134(2)	0.0109(2)	0	0	0.0007(2)
K3	0.0115(2)	0.0115(2)	0.0251(4)	0	0	0
Na1	0.0130(4)	0.0130(4)	0.0162(8)	0	0	0
Si1	0.0033(2)	0.0036(2)	0.0014(2)	0.0001(2)	0.0000(2)	-0.0002(2)
Si2	0.0061(2)	0.0043(2)	0.0018(2)	-0.0005(2)	-0.0003(2)	0.0026(2)
O1	0.0041(5)	0.0072(5)	0.0042(5)	-0.0002(4)	0.0001(4)	-0.0024(4)
O2	0.0148(6)	0.0056(5)	0.0025(5)	0.0007(4)	-0.0004(4)	0.0020(4)
O3	0.0075(5)	0.0044(5)	0.0052(5)	0.0004(4)	0.0010(4)	-0.0002(4)
O4	0.0057(5)	0.0121(6)	0.0026(5)	-0.0011(4)	0.0000(4)	0.0010(4)
O5	0.0051(5)	0.0139(6)	0.0025(5)	0.0010(4)	-0.0003(4)	0.0006(4)
O6	0.0071(5)	0.0048(5)	0.0045(5)	0.0001(4)	0.0004(4)	0.0018(4)
O7	0.0124(6)	0.0064(5)	0.0026(5)	-0.0008(4)	-0.0007(4)	0.0016(4)
O8	0.0066(5)	0.0183(6)	0.0050(5)	-0.0010(4)	0.0001(4)	0.0056(5)
Li1	0.019(2)	0.008(2)	0.004(2)	0.001(2)	0.000(2)	0.002(2)
Li2	0.007(2)	0.011(2)	0.005(2)	0.001(2)	0.000(1)	-0.001(2)
Li3	0.015(2)	0.007(2)	0.007(2)	-0.001(2)	-0.001(2)	0.000(2)
Li4	0.023(2)	0.008(2)	0.006(2)	-0.000(2)	-0.003(2)	0.005(2)
Li5	0.019(2)	0.009(2)	0.007(2)	-0.000(2)	-0.001(2)	0.005(2)
Li6	0.012(2)	0.053(3)	0.005(2)	-0.002(2)	0.001(2)	-0.016(2)

Table S4. Interatomic distances (pm)

K1–O6	262.6(2)	K2–O3	272.0(2) 2×	K3–O7	272.6(2) 4*	Na1–O3	256.0(2) 4*
–O8	267.7(2)	–O1	279.0(2) 2*	–O2	277.9(2) 4*	–O1	270.7(2) 4*
–O5	268.5(2)	–O7	280.4(2) 2*	**Ø**	**275.3**	**Ø**	**263.3**
–O6	274.4(2)	–O2	289.8(2) 2*				
–O4	281.1(2)	–O5	345.8(2) 2*				
–O4	283.9(2)	**Ø**	**280.3**				
–O8	287.4(2)						
–O5	340.3(2)						
**Ø**	**283.2**						
Si1–O5	163.5(2)	Si2–O8	162.9(2)	Li1–O5	192.7(4)	Li2–O1	189.9(4)
–O4	163.6(2)	–O2	163.8(2)	–O7	193.5(4)	–O8	195.0(4)
–O3	163.9(2)	–O7	164.0(2)	–O6	197.0(4)	–O4	202.1(4)
–O1	164.0(2)	–O6	165.4(2)	–O2	213.9(4)	–O3	204.1(4)
**Ø**	**163.7**	**Ø**	**164.0**	**Ø**	**199.3**	**Ø**	**197.8**
Li3–O8	191.9(4)	Li4–O5	189.2(4)	Li5–O6	192.7(4)	Li6–O4	190.6(4)
–O2	192.3(4)	–O3	191.7(4)	–O2	193.7(4)	–O7	194.0(4)
–O6	197.6(4)	–O1	193.6(4)	–O1	198.6(4)	–O3	197.6(4)
–O7	214.9(4)	–O4	233.4(5)	–O5	225.0(4)	–O8	257.0(5)
**Ø**	**199.1**	**Ø**	**202.0**	**Ø**	**202.5**	**Ø**	**209.8**

Charge distribution and Madelungs part of lattice energy calculations

Charge distributions were calculated according to both the bond-length/bond-strength (∑V)[7-8] and the CHARDI (∑Q)[9] concept (Table S5). Within the limits of these concepts, the calculated charges correlate well with the expected values.

Table S5. Interatomic distances (pm)

	**K1**	**K2**	**K3**	**Na1**	**Si1**	**Si2**
**∑V**	+1.34	+1.32	+1.50	+0.86	+3.86	+3.82
**∑Q**	+1.02	+0.97	+1.00	+0.95	+4.02	+4.01

	**Li1**	**Li2**	**Li3**	**Li4**	**Li5**	**Li6**
**∑V**	+0.99	+1.02	+1.00	+0.99	+0.93	+0.89
**∑Q**	+1.00	+1.01	+1.00	+0.99	+0.97	+1.01

	**O1**	**O2**	**O3**	**O4**	**O5**	**O6**	**O7**	**O8**
**∑V**	-2.05	-2.00	-2.05	-1.90	-1.92	-2.05	-2.04	-1.96
**∑Q**	-2.11	-1.99	-2.11	-1.86	-1.92	-2.12	-2.02	-1.89

The MAPLE value (Madelung part of lattice energy)[10-12] of $NaK_7[Li_3SiO_4]_8$ was calculated and compared to the value obtained from the summation of the MAPLE values of the oxides Na_2O, K_2O, Li_2O, and SiO_2. The values are in good agreement ($NaK_7[Li_3SiO_4]_8$: 174548 kJ mol^{-1}; 0.5 Na_2O + 3.5 K_2O + 12 Li_2O + 8 SiO_2: 174385 kJ mol^{-1}) and deviate by less than 0.1 %.

Table S6. Comparison of the calculated Maple values of $NaK_7[Li_3SiO_4]_8$ and the oxide species Na_2O, K_2O, Li_2O, and SiO_2.

calculated Maple value for $NaK_7[Li_3SiO_4]_8$ in kJ mol^{-1}	174548
calculated Maple value for the educt compounds in kJ mol^{-1}	174385
deviation in %	0.01

Extended Coordination of the Alkalimetal-Cations

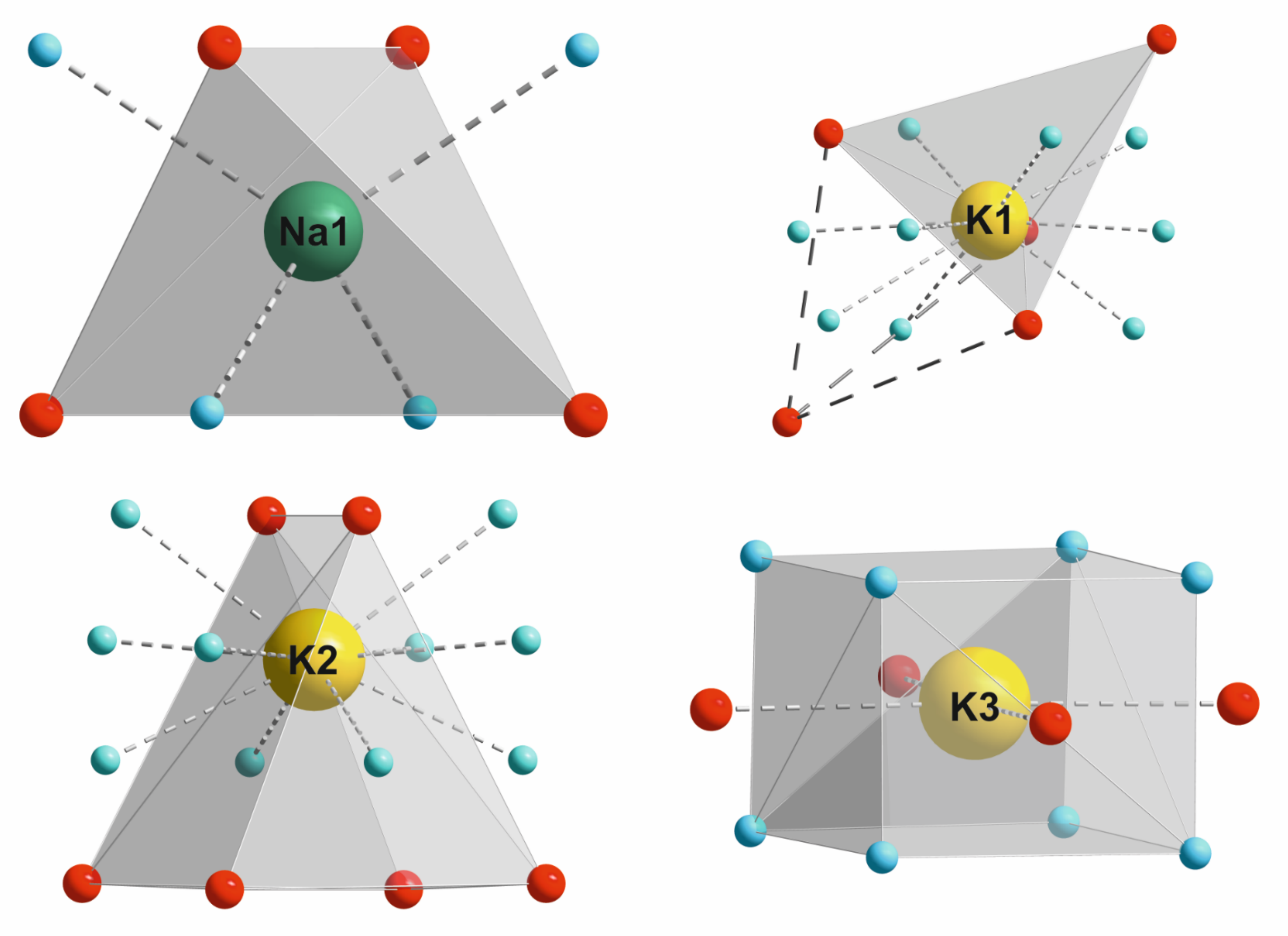

Figure S1. Extended coordination spheres of the Na1, K1, K2, and K3 sites towards the silicon (red) and lithium (blue) cations.

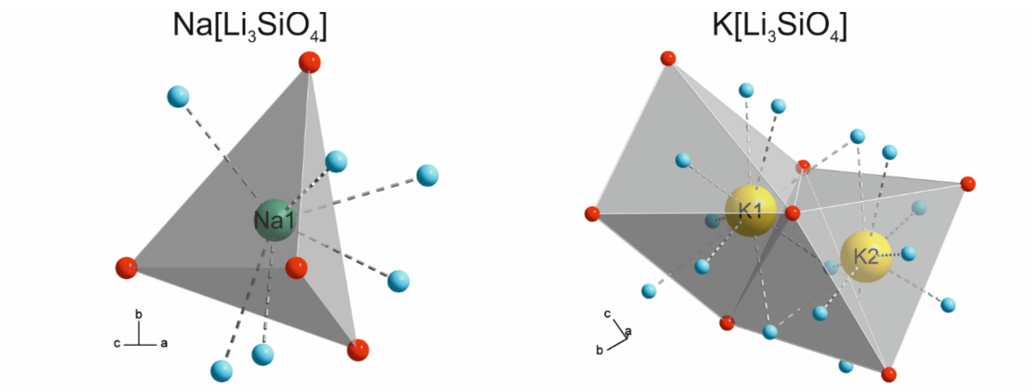

Figure S2. Extended coordination spheres of sodium in Na[Li$_3$SiO$_4$] (left), and potassium K[Li$_3$SiO$_4$] (right) towards silicon (red) and lithium (blue).

Excitation spectra

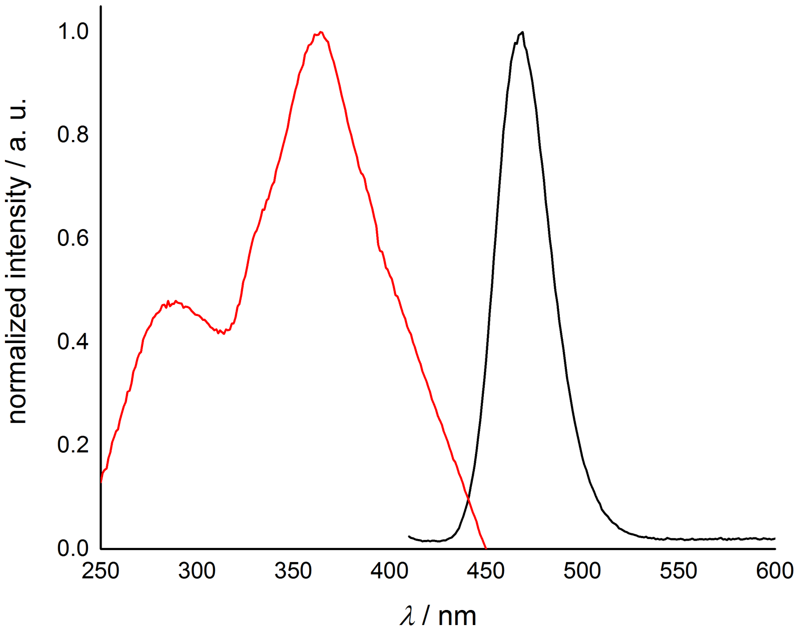

Figure S3. Excitation spectrum monitored at λ_{em} = 467 nm (red) and emission spectrum with λ_{exc} = 400 nm (black) of $Na[Li_3SiO_4]:Eu^{2+}$.

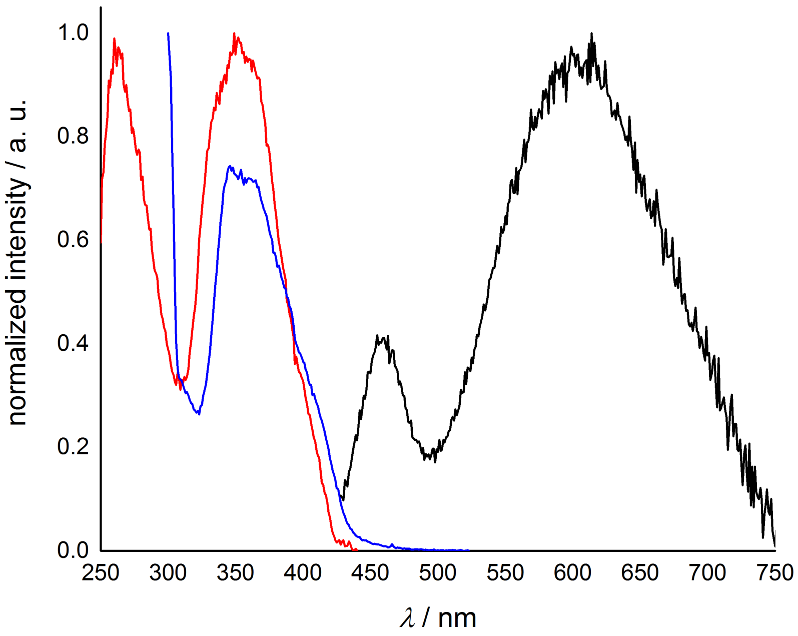

Figure S4. Excitation spectrum monitored at λ_{em} = 457 nm (red) and at λ_{em} = 580 nm (blue), emission spectrum with λ_{exc} = 400 nm (black) for the double-band emission of $K[Li_3SiO_4]:Eu^{2+}$.

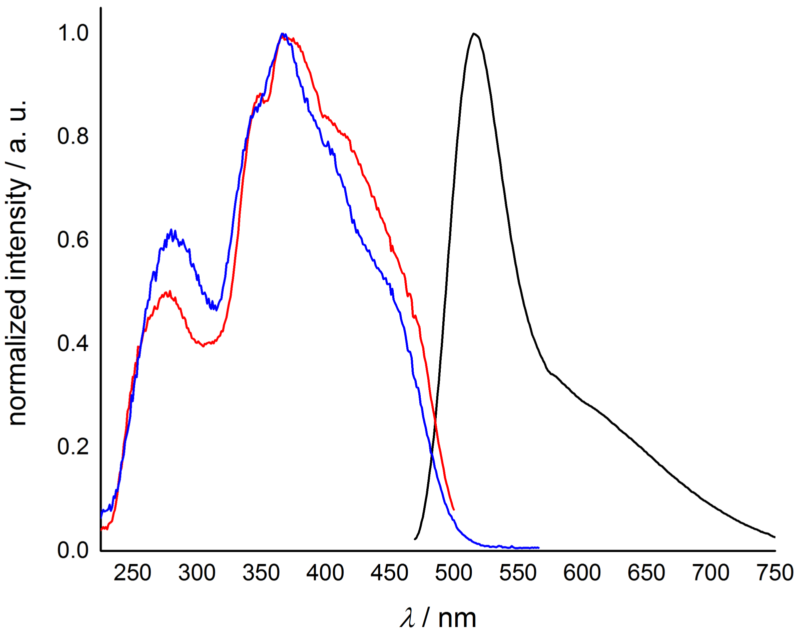

Figure S5. Excitation spectrum monitored at λ_{em} = 515 nm (blue) and at λ_{em} = 598 nm (red), emission spectrum with λ_{exc} = 460 nm (black) for the double-band emission of $NaK_7[Li_3SiO_4]_8:Eu^{2+}$.

Thermal Quenching (TQ)

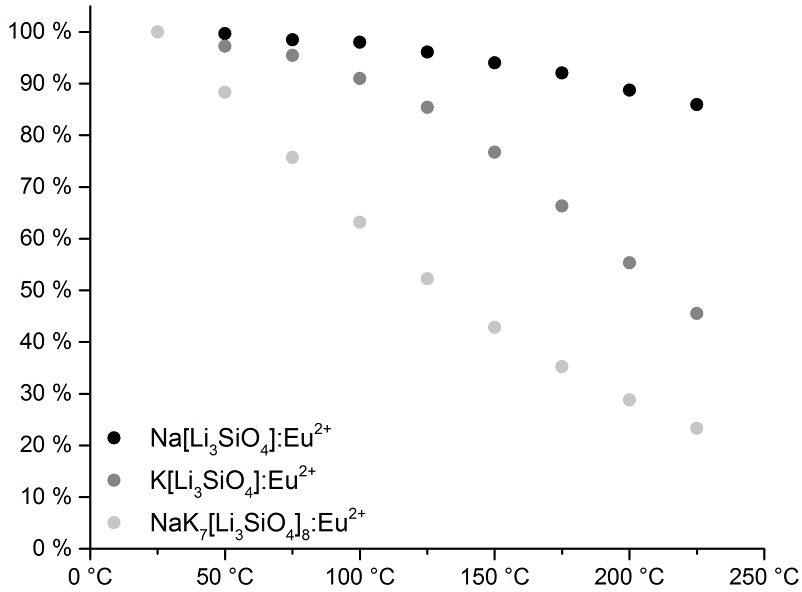

Figure S6. Comparison of the thermal quenching properties of $Na[Li_3SiO_4]:Eu^{2+}$ (black), $K[Li_3SiO_4]:Eu^{2+}$ (gray), and $NaK_7[Li_3SiO_4]_8:Eu^{2+}$ (light gray) in the range of 25 °C to 225 °C.

Quantum efficiency (QE)

Table S7. Comparison of the quantum efficiency of $Na[Li_3SiO_4]:Eu^{2+}$, $K[Li_3SiO_4]:Eu^{2+}$, and $NaK_7[Li_3SiO_4]_8:Eu^{2+}$.

	QE
$Na[Li_3SiO_4]:Eu^{2+}$	65 %
$K[Li_3SiO_4]:Eu^{2+}$	57 %
$NaK_7[Li_3SiO_4]_8:Eu^{2+}$	54 %

References

[1] B. Nowitzki, R. Hoppe, *Rev. Chim. Minér.* **1986**, *23*, 217–230.
[2] Bruker *SAINT*, v8.34a, Bruker AXS Inc., Madison, WI, USA: 2014.
[3] G. M. Sheldrick, SADABS, v2014/5, Bruker AXS Inc., Madison, WI, USA: 2001.
[4] G. Sheldrick, *Acta Crystallogr., Sect. A* **2008**, *64*, 112–122.
[5] G. M. Sheldrick, *Acta Crystallogr., Sect. C* **2015**, *71*, 3–8.
[6] L. Farrugia, *J. Appl. Crystallogr.* **2012**, *45*, 849–854.
[7] I. D. Brown, D. Altermatt, *Acta Crystallogr., Sect. B* **1985**, *41*, 244–247.
[8] I. D. Brown, in *Bond Valences. Structure and Bonding, Vol. 158* (Eds.: I. D. Brown, P. K), Springer, Berlin, Heidelberg, **2013**, pp. 11–58.
[9] R. Hoppe, S. Voigt, H. Glaum, J. Kissel, H. P. Müller, K. Bernet, *J. Less-Common Met.* **1989**, *156*, 105–122.
[10] R. Hoppe, *Angew. Chem. Int. Ed.* **1966**, *5*, 95–106.
[11] R. Hoppe, *Angew. Chem. Int. Ed.* **1970**, *9*, 25–34.
[12] R. Hübenthal, *MAPLE*, v4, University of Gießen, Germany: 1993.

Author Contributions

H.H. and D.D. coordinated the research and wrote the main parts of the manuscript. D.D. performed the syntheses and solved the crystal structure of $NaK_7[Li_3SiO_4]_4$. M.S. and D.B. performed the investigations regarding the luminescence properties. D.D., M.S., D.B. co-wrote the manuscript. All authors commented on the paper.
